# Intestinal and Blood–Brain Barrier Dysfunction in Lupus: Emerging Mechanisms and Modulation by Cinnamon

**DOI:** 10.3390/molecules31142514

**Published:** 2026-07-18

**Authors:** Georges Maalouly, Georges-Alain Al Tekle, Grisselle Al Achkar, Nassim Fares

**Affiliations:** 1 Laboratory of Research in Physiology and Pathophysiology, Faculty of Medicine, Saint Joseph University of Beirut, Beirut 1104 2020, Lebanon; 2 Faculty of Medicine, Saint Joseph University of Beirut, Beirut 1104 2020, Lebanon; georgesalain.tekle@net.usj.edu.lb (G.-A.A.T.); grisselle.achkar@net.usj.edu.lb (G.A.A.)

**Keywords:** lupus, natural compounds, tight junction, gut permeability, blood–brain barrier, cinnamon

## Abstract

Systemic lupus erythematosus (SLE) is a complex autoimmune disease with evolving pathogenesis. Biological barriers, especially intestinal and blood–brain barriers (BBBs) with their tight junctions (TJ), are gaining attention in recent years as key players in disease initiation and progression. Among natural products targeting these barriers, cinnamon is emerging as a multi-targeted modulator of TJ. This narrative review integrates current evidence about gut and brain barrier dysfunction in lupus pathogenesis and highlights, on the basis of animal studies, the potential of cinnamon as a therapeutic candidate to restore barrier integrity and attenuate immune and neuroinflammation associated with lupus. Experimental evidence from lupus models supports the role of TJ disruption in disease pathogenesis. The alteration of TJ protein distribution in the epithelial barrier is correlated with an increased permeability of the intestinal barrier and changes in the microbiota composition in lupus, with consequent alteration in the gut–liver axis, liver inflammation and oxidative stress. Pre-clinical studies have demonstrated the restorative effect of cinnamon on gut TJ and permeability, microbiota and the gut–liver axis. Moreover, accumulating data suggest BBB disruption in lupus, correlated with neuroinflammation and behavioral disturbances. A murine model demonstrates the protective effect of cinnamon on BBB, especially via TJ localization, with the alleviation of neuropsychiatric alterations. Future perspectives should focus on cinnamon’s effect on the gut–brain axis and translational studies.

## 1. Introduction

The complex and multi-faceted nature of systemic lupus erythematosus (SLE) necessitates a comprehensive understanding of the interplay between systemic inflammation and organ-specific damage [1]. Although SLE has been traditionally viewed through the lens of immune complex deposition and the production of nuclear autoantibodies, recent advances in mucosal immunology and neurobiology have highlighted the critical role of biological barriers in the progression of the disease [2,3]. Accumulating data highlight the critical role of TJ in maintaining the integrity of barrier systems, in particular the intestinal epithelium and blood–brain barrier (BBB), which may be compromised in lupus patients [4,5]. Elucidating the molecular mechanisms of barrier dysfunction in lupus is essential in order to understand their contribution to disease initiation and progression and to identify novel therapeutic strategies.

The intestinal barrier functions as a selective barrier, preventing harmful substances from entering the bloodstream while allowing nutrients to pass through [6]. When compromised, this barrier can lead to systemic inflammation, immune dysregulation, exacerbated autoimmune diseases and a disrupted gut–brain axis [7,8,9]. Studies have highlighted increased intestinal permeability in lupus, suggesting a potential link to altered tight junction protein expression [10].

The BBB plays an essential role in maintaining the central nervous system’s (CNS) homeostasis [11]. Recent investigations have highlighted various inflammatory mediators, including complement components and pro-inflammatory cytokines, that undermine BBB integrity, leading to neuroinflammation and the exacerbation of psychiatric diseases [12,13]. The degradation and dysfunction of TJ proteins have been implicated in the pathogenesis of neuropsychiatric SLE (NPSLE), where neuroinflammation and BBB disruption contribute to the clinical manifestations of the disease [14,15]. Additionally, the neuropsychiatric aspects of lupus have been associated with the signaling pathways known to affect the BBB’s integrity by influencing TJ permeability [16].

In the context of TJs and biological barrier regulation, the exploration of natural compounds has gained significant attention due to their potential therapeutic effects on various health conditions [17]. Key bioactive components, such as flavonoids and α,β-unsaturated compounds, can influence the opening and function of TJ, modulating permeability [18]. From promoting intestinal regeneration and mitigating neuroinflammation to providing novel approaches to cancer treatment, these compounds exhibit multi-faceted bioactivity that warrants further investigation [19].

As a widely used natural compound, cinnamon has garnered attention in recent research for its potential therapeutic effects, particularly in relation to tight junctions (TJ), which are critical for maintaining epithelial barrier integrity. In vitro studies using Caco-2 cells demonstrated that cinnamon hot water extract improved the transepithelial electrical resistance, which is indicative of enhanced TJ function [20,21]. Additionally, recent studies have highlighted the potential of cinnamon to modulate neuronal inflammation and protect against BBB compromise [22,23].

This narrative review integrates current evidence to elucidate the gut and brain barrier dysfunction in lupus pathogenesis and to highlight the potential of cinnamon as an emerging modulator capable of restoring barrier integrity and attenuating immune and neuroinflammation associated with lupus.

## 2. Lupus Evolving Pathogenesis

SLE is the classic prototype of autoimmune diseases, characterized by a vast spectrum of clinical manifestations and significant phenotypic variability [24]. In genetically and epigenetically predisposed individuals, the pathogenesis is initiated when autoantigens, derived from neutrophil NETosis and rapid cellular turnover, overwhelm the innate immune system’s regulated clearance mechanisms [25]. These accumulated autoantigens trigger Toll-like receptor (TLR) signaling within plasmacytoid dendritic cells (pDCs), leading to a massive production of Type 1 Interferon (IFN) [26]. This interferon signature sensitizes both innate and adaptive immune branches, activating autoreactive T and B cells and culminating in the production of pathogenic immune complexes [27,28]. As these complexes deposit in various tissues, they incite localized inflammatory responses that, through recurrent flares, eventually transition from attempted repair to permanent fibrosis [29,30].

Recent studies have further refined this model, highlighting the primordial role of innate triggers such as TLR7 and environmental catalysts. In 2022, Brown et al. identified a TLR7 gene variant in a pediatric case that, when replicated in experimental models, fully recapitulated lupus symptoms by recruiting MyD88 to activate IRF7 and NF-κB, driving both IFN and pro-inflammatory cytokine production [31]. This genetic susceptibility is often activated by environmental factors; notably, a study by Younis et al. in late 2025 provided further evidence for the causal role of the Epstein–Barr Virus (EBV) in SLE [32]. Furthermore, oxidative stress and immunometabolism act as a critical regulator for the disease, where an imbalance of reactive oxygen species (ROS) damages DNA and lipids and create neo-autoantigens. These modified molecules further stimulate TLR7, fueling a vicious cycle of chronic inflammation and organ damage, particularly within the kidneys as lupus nephritis [33].

To navigate this biological complexity, researchers utilize systems biology and multi-omics technologies to integrate multifaceted data into coherent diagnostic and therapeutic frameworks [34,35]. Animal models have highlighted different aspects of the disease: spontaneous mutant strains allow for the dissection of specific genetic contributions, while induced models are uniquely positioned to highlight environmental influences and the disease’s temporal evolution [36,37]. Despite these advances, a significant gap remains in clinical practice regarding the role of diet in the prevention and treatment of lupus. Current recommendations focus on corticosteroids toxicities, the role of immunosuppression and targeted therapy and associated infectious and metabolic risks [38]. While nutritional interventions are not fully detailed in international management guidelines, they represent a promising research frontier [39,40]. Major interfaces between natural interventions and the dynamic of lupus pathogenesis are biological barriers, especially the gut barrier and BBB [41,42].

## 3. Cinnamon: A Spice with Multiple Targets

Cinnamon, a popular and widely used spice, has attracted attention in various medical research fields for its potential effects on multiple diseases. The use of network pharmacology enabled the identification of various targets and pathways involved, enhancing the understanding of the multi-target effects of cinnamon components. Cinnamon and its derivatives exhibit substantial pharmacological potentials, including anti-inflammatory, anti-diabetic, and hepatoprotective effects [43,44]. Network pharmacology and experimental studies have highlighted mechanisms involving various signaling pathways such as TLR4, TLR7, JAK-STAT and PI3K/Akt, along with antioxidant properties [15,45,46].

Cinnamon is composed of many compounds with biological activities. Cinnamaldehyde is the basic compound found in the cinnamon bark. Cinnamaldehyde is metabolized mainly by the oxidation of its aldehyde group to cinnamic acid [47]. In the liver, cinnamic acid undergoes mitochondrial beta-oxidation to yield benzoic acid (or its active intermediate, benzoyl-CoA) [48]. Other bioactive compounds of cinnamon are procyanidin polymer type A, cinnamic acid, and coumarin. The amount of the constituents depend on the method of separation, extraction, and purification [49].

**Figure 1:**
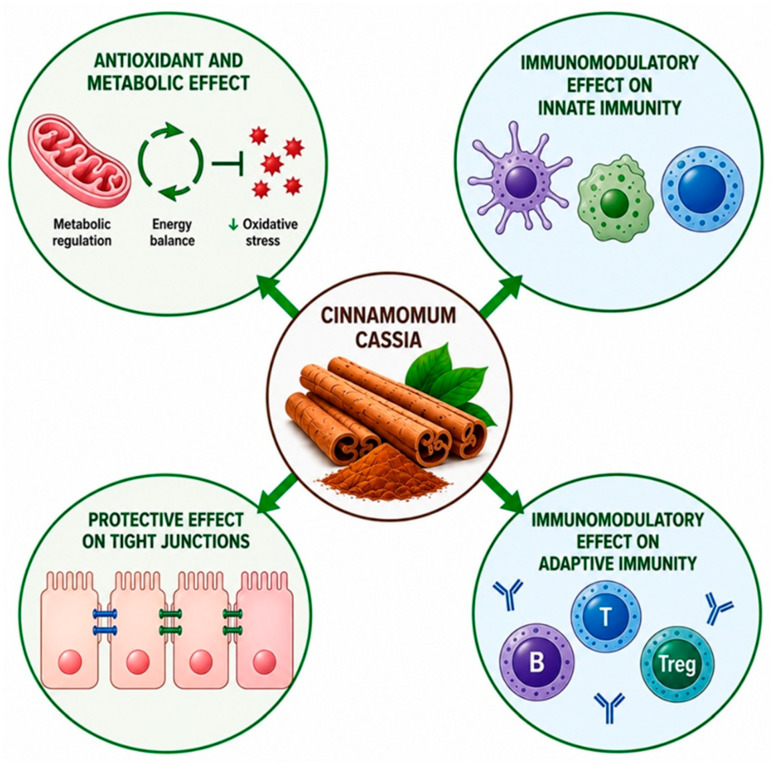
Multi-biological targets of *Cinnamomon cassia*.

The complex mechanisms underlying autoimmune conditions and the natural properties of cinnamon have led researchers to investigate whether this spice may play a therapeutic role in managing metabolic and autoimmune disorders [50] (Figure 1).

Cinnamaldehyde, an active component of cinnamon, has been studied for its specific anti-inflammatory effects, including the suppression of NLRP3 inflammasome activation, which is pivotal in various autoimmune processes [51]. The cinnamon metabolites cinnamic acid and sodium benzoate have been shown to enhance regulatory T cell populations and upregulate TGF-β, which can confer protection against autoimmune conditions such as multiple sclerosis [52,53]. Cinnamon (especially cinnamaldehyde) has also been analyzed in the context of rheumatoid arthritis, where it exhibited a positive effect on reducing disease activity scores and inflammatory markers [54]. Additionally, cinnamon’s influence on metabolic processes (especially *Cinnamomum verum* bark extracts) may contribute to its antidiabetic effects, highlighting its multifaceted role in treating conditions associated with immune dysregulation in diabetic patients [45].

In parallel, cinnamon and its derivatives demonstrate significant effects on TJ physiology in intestinal epithelium and BBB. Cinnamon subcritical water extract was shown to enhance the expression of TJ proteins in a cellular intestinal inflammation model, improving intestinal barrier function and reducing inflammation by inhibiting key inflammatory agents like NF-κB and TNF-α [20]. Another investigation into *Cinnamomum cassia* hot water extract highlighted its effectiveness in improving tight junction integrity and reducing inflammation in both in vitro and in vivo models of colitis [21]. Moreover, cinnamon hydroalcoholic extract reduced blood–brain barrier (BBB) permeability by increasing the expression of TJ proteins in a rat model of stroke while down-regulating pro-inflammatory markers such as calpain and VEGF [23].

## 4. Gut Permeability in Lupus and Modulation by Cinnamon

The intestinal barrier integrates epithelial tight junctions (TJs), mucus, immune defenses, and microbiota to permit selective absorption while limiting microbial translocation [55]. In lupus-relevant models, barrier loss is linked to endotoxaemia, bacterial translocation to mesenteric lymph nodes and the liver, and the amplification of autoimmunity [56,57]. In experimental lupus animal models, natural compound supplementation was associated with the modulation of intestinal TJ proteins and possibly gut–liver axis activation homeostasis [58,59].

### 4.1. TJ as Gatekeepers of the Gut Barrier

The intestinal barrier is a dynamic interface that constrains systemic immunological and physiological exposure to luminal microbes and microbial ligands while enabling nutrient uptake. When compromised, increased permeability and translocation can provide persistent stimuli that shape immunity and potentially amplify autoimmune disease activity [57]. The barrier comprises multiple integrated layers: (i) microbiota and their metabolites, (ii) mucus and antimicrobial products, (iii) the epithelial monolayer sealed by apical junctional complexes, and (iv) mucosal immune surveillance. TJs organized around claudins, occludin, and scaffold proteins such as ZO-1 are dynamic regulators of paracellular flux and respond to cytokines, microbial cues, and cytoskeletal tension [57]. Barrier functionality depends on permeability flux, epithelial integrity, tight junction abundance and localization, and inflammatory markers such as calprotectin. Barrier integrity can improve through TJ localisation and assembly even if total protein levels change modestly, as shown in epithelial cinnamaldehyde studies [60]. Barrier dysfunction stems from multiple pathobiological mechanisms: cytokine-driven TJ alteration, epithelial oxidative stress, apoptosis, and mucus layer disruption; this imbalance increases permeability, further fueling microbial translocation and immune activation [57].

### 4.2. Key Evidence in Lupus

Experimental evidence from lupus murine models further supports the role of TJ disruption in disease pathogenesis. The alteration of TJ protein distribution in the epithelial barrier is correlated with an increased permeability of the intestinal barrier and changes in the microbiota composition in a lupus model using transgenic mice overexpressing TLR7 [61]. The reduced expression of the TJ protein Claudin-1 was also observed in R848-treated lupus-prone mice [62]. Raised serum levels of zonulin, a regulator of TJ formation between cells that form the gut barrier, were induced in mice by *Blautia* (*Ruminococcus*) *gnavus* strains isolated from lupus patients in a human population [63].

### 4.3. Mechanisms Involved

Inflammation plays a central role in TJ disruption and increased intestinal permeability in lupus. Pro-inflammatory cytokines such as TNF-α and IFN-γ alter the expression and localization of TJ proteins and promote cytoskeletal rearrangement, leading to barrier dysfunction [64]. These cytokines activate intracellular signaling pathways, particularly NF-κB, which induces myosin light chain kinase (MLCK) activation and increases paracellular permeability through the contraction of the actomyosin cytoskeleton [65]. In addition, gut dysbiosis contributes to barrier impairment by promoting inflammatory signaling and immune activation. Zonulin further regulates intestinal permeability by modulating TJ opening, and its dysregulation is associated with increased permeability and autoimmune processes [66].

### 4.4. Lupus Consequences

Leaky gut promotes autoantibody generation, including anti-dsDNA IgG, by allowing commensal antigens to trigger systemic immune responses and worsen inflammation and organ involvement in lupus [67]. This barrier dysfunction, combined with dysbiosis, alters mucosal immune responses such as IgA regulation, further contributing to systemic autoimmunity, as observed in human studies [68]. Maalouly et al. demonstrated that intestinal barrier impairment in murine imiquimod-induced lupus leads to a significant translocation of *E. coli* proteins into the liver, indicating the disruption of the gut–liver axis and the penetration of bacterial products. These microbial components activate Toll-like receptors, including TLR7, which recruit adaptor proteins such as MyD88 and trigger NF-κB signalling, resulting in the increased production of pro-inflammatory cytokines like IL-6 and TNF-α [69]. Chronic hepatic inflammation is associated with oxidative stress and redox imbalance, characterized by increased reactive oxygen species and the compensatory upregulation of antioxidant enzymes such as SOD1 and SOD2, as observed in lupus animal models and modulated by interventions targeting the gut–liver axis [70]. These findings highlight the central role of intestinal barrier dysfunction and microbial translocation in lupus pathogenesis, thereby supporting the investigation of therapeutic strategies targeting the gut–liver axis.

### 4.5. Modulation by Cinnamon

*Cinnamomum cassia* supplementation modulates key elements of the gut–liver axis in murine lupus by influencing microbial ecology and host responses without significantly altering overall microbiota diversity, highlighting a targeted rather than global microbiome effect. In imiquimod-induced murine lupus, cinnamon (powder of *Cinnamomum cassia* in the form of the powder of cinnamon bark with bark extract) mitigated the disease-associated trend toward a reduced Firmicutes/Bacteroidota ratio and decreased the abundance of Lachnospiraceae, suggesting the selective modulation of microbial composition and the restoration of microbial balance [70]. Importantly, cinnamon effectively reverses the increase in *E. coli* protein detected in the liver, reflecting reduced microbial translocation and improved intestinal barrier integrity, a key feature of gut–liver axis dysfunction in lupus [69]. This improvement is accompanied by the normalization of hepatic inflammatory pathways, including decreased activation of TLR-7 and NF-κB signalling, along with the reduced expression of antioxidant enzymes SOD1 and SOD2, indicating the attenuation of both inflammatory and oxidative stress responses [70] (see Table 1). Together, these findings position cinnamon as a multi-mechanistic modulator of lupus pathology, acting on microbial dysbiosis, intestinal permeability, and downstream inflammatory and oxidative pathways within the gut–liver axis in experimental studies [70].

## 5. BBB in Lupus and Modulation by Cinnamon

The BBB plays a key role in maintaining central nervous system homeostasis by regulating the passage of molecules between the circulation and the brain. In SLE, its disruption has been increasingly linked to neuropsychiatric manifestations, as it allows peripheral inflammatory mediators and autoantibodies to access neural tissue [71,72].

This dysfunction is thought to result from inflammatory and oxidative processes that impair endothelial integrity and promote neuroinflammation [72]. Interest has grown in natural compounds that may modulate these alterations [73,74]. Among them, cinnamon derivatives have been investigated for their potential neuroprotective effects, particularly through their anti-inflammatory and antioxidant properties [75,76].

### 5.1. TJs as Gatekeepers of BBB

TJs play a crucial role in maintaining the integrity of the BBB, restricting paracellular diffusion, and safeguarding the central nervous system from potentially harmful substances in the bloodstream [77]. Various proteins, including claudins, occludin, and zonula occludens (ZO) proteins, contribute to this function. Morphologically, these TJs are closer to epithelial tight junctions than endothelial ones typically found in peripheral vasculature [78]. Moreover, they engage in signaling pathways that affect cytoskeletal organization and cellular responses to environmental changes [77]. Pathophysiological conditions, such as ischemic stroke and inflammatory responses, can alter the integrity of the BBB through the modulation of TJ proteins, leading to increased permeability [79,80]. Tight junction protein expression within the BBB exhibits high molecular heterogeneity. While the standard endothelial phenotype is characterized by the presence of claudin-5, -12, and -25, the upregulation of claudin-1 is observed in response to altered environmental stimuli or experimental interventions [81].

### 5.2. Key Evidence in Lupus

Converging evidence from neuroimaging, biomarker analyses, and experimental models supports the involvement of BBB dysfunction in neuropsychiatric systemic lupus erythematosus (NPSLE). Advanced imaging approaches, particularly dynamic contrast-enhanced magnetic resonance imaging (DCE-MRI), have demonstrated increased BBB permeability, often quantified using the Ktrans parameter, in human patients with SLE [82]. This alteration has been associated with cognitive impairment and structural brain changes, including gray matter loss [83]. Importantly, increased BBB permeability has also been linked to psychiatric manifestations, including depression and anxiety, further highlighting the broad neuropsychiatric impact of barrier dysfunction in SLE induced in genetic background mice [84].

In parallel, clinical investigations suggest that BBB leakage contributes to neurological dysfunction independently of circulating autoantibody levels, underscoring the role of barrier integrity as a key determinant of central nervous system involvement rather than a mere consequence of systemic immune activity in lupus patients [71]. Complementing these findings, circulating and cerebrospinal fluid biomarkers provide additional insight into CNS injury. Elevated levels of glial fibrillary acidic protein (GFAP) have been associated with disease severity and central nervous system injury [85]. Similarly, increased neurofilament light chain (NfL) levels have been linked to neuronal damage and poorer neurological outcomes in human studies [86,87].

Experimental models further strengthen this association. In lupus-prone mice such as MRL/lpr and NZB/W F1 strains, BBB disruption occurs early in disease progression and parallels the development of neurobehavioral abnormalities. In MRL/lpr mice, increased permeability facilitates the entry of autoreactive antibodies into the brain, leading to neuronal damage and psychiatric manifestations [5]. Similarly, NZB/W F1 mice exhibit neurological deficits associated with immune-mediated alterations of BBB integrity [5]. Importantly, several studies suggest that BBB dysfunction may precede overt neuropsychiatric symptoms, particularly in regions such as the hippocampus, supporting its role as an early pathogenic event [5]. In addition, the experimental disruption of the BBB has been shown to induce anxiety- and depression-like behaviors, further reinforcing the causal link between barrier breakdown and central nervous system dysfunction [41].

Furthermore, experimental studies indicate that BBB dysfunction may be modifiable. In imiquimod-induced lupus, which relies on the activation of Toll-like receptor 7 (TLR7), neuropsychiatric features including cognitive impairment, behavioral alterations, and brain lesions have been described in wild-type mice [88].

Taken together, these findings indicate that BBB dysfunction is not merely a secondary phenomenon but rather a central component linking systemic autoimmunity to neurological manifestations in SLE.

### 5.3. Disruption Mechanisms

BBB disruption in SLE arises from a combination of inflammatory and immune-mediated processes that collectively impair endothelial integrity. Inflammatory cytokines, complement activation, oxidative stress, and autoantibody-mediated effects converge to increase barrier permeability, thereby facilitating immune cell infiltration and promoting neuroinflammation [89].

In this context, innate immune signaling pathways such as Toll-like receptor 7 (TLR7) are increasingly recognized as potential upstream contributors to neuroinflammation. Early evidence has implicated TLR7 signaling in inflammatory processes within the central nervous system [90]. More recent work has linked this pathway to oxidative stress and the activation of inflammasome signaling, including NLRP3, further supporting its role in amplifying neuroinflammatory cascades [91]. However, its direct contribution to blood–brain barrier disruption remains to be fully established.

Among the contributing mechanisms, complement activation, particularly through the generation of C5a, plays a central role by inducing endothelial injury and apoptosis, which weaken the structural stability of the barrier [92,93]. In parallel, elevated levels of pro-inflammatory cytokines, including interleukin-6 (IL-6), interleukin-18 (IL-18), and tumor necrosis factor-alpha (TNF-α), sustain a pro-inflammatory milieu that disrupts TJ organization. These cytokines promote the upregulation of matrix metalloproteinases (MMPs), leading to the degradation of extracellular matrix components and the reduced expression of TJ proteins such as claudin-5 and occludin, ultimately increasing BBB permeability [72].

In addition, inflammatory signaling enhances the expression of adhesion molecules such as ICAM-1 and VCAM-1 on endothelial cells, facilitating leukocyte adhesion and transmigration across the BBB, thereby amplifying central nervous system inflammation [94]. As barrier integrity deteriorates, circulating autoantibodies gain access to the central nervous system. Among the most extensively studied are anti-N-methyl-D-aspartate (NMDA) receptor antibodies and anti-ribosomal P antibodies, which can cross-react with neuronal targets and induce excitotoxic injury [95]. At the same time, chemokines such as CCL2 promote the recruitment of peripheral immune cells into the CNS, further amplifying the inflammatory response [72].

Microglial activation represents a key amplification step in this cascade. Once activated, microglia adopt a pro-inflammatory phenotype, releasing cytokines and reactive mediators that contribute to synaptic remodeling, the phagocytosis of neuronal elements, and progressive neuronal loss, thereby sustaining neuroinflammation [72].

Additional pathways have also been implicated in BBB disruption. The TWEAK/Fn14 signaling axis has been associated with endothelial activation and the increased production of pro-inflammatory mediators [96]. It has also been linked to the upregulation of matrix metalloproteinases, contributing to increased permeability and immune cell infiltration [5].

In addition to the classical BBB, a dysfunction of the blood–cerebrospinal fluid (CSF) barrier at the level of the choroid plexus has been proposed to occur early in the disease process, potentially allowing the initial entry of inflammatory mediators and autoantibodies into the CNS [97].

### 5.4. Lupus Consequences

The disruption of the BBB permits the entry of peripheral immune mediators and cells into the central nervous system, initiating and sustaining neuroinflammation and neuronal dysfunction [72]. This increased BBB permeability has been associated with the accumulation of inflammatory mediators that can interfere with neuronal signaling and contribute to brain injury [98]. In addition, alterations in TJ integrity and endothelial function facilitate leukocyte transmigration, further amplifying inflammatory responses within the central nervous system [94,99].

These processes are associated with impaired hippocampal neurogenesis, characterized by the increased apoptosis of neural stem cells and reduced neuronal differentiation, ultimately contributing to neuronal loss. The hippocampus appears to be particularly vulnerable in neuropsychiatric lupus and represents a key target of immune-mediated injury [100]. Experimental studies have also reported the deposition of immunoglobulin G (IgG) within hippocampal tissue, reflecting the increased permeability of the BBB and local immune activation [101]. This deposition has been associated with microglial activation toward a pro-inflammatory phenotype, further contributing to neuronal dysfunction [72]. Similar findings have been reported in imiquimod-induced lupus, where hippocampal injury—characterized by neuronal apoptosis and IgG deposition—has been observed in parallel with behavioral alterations reflecting anxiety, depressive, and cognitive-like features [3].

Moreover, excessive exposure to inflammatory mediators and neuroactive substances can disrupt synaptic function and neuronal communication. Clinically, these alterations manifest as a spectrum of neuropsychiatric symptoms, including anxiety, depression, cognitive impairment, and, in severe cases, psychosis [95]. In this context, the persistent activation of innate immune pathways, including Toll-like receptor 7 (TLR7), may further amplify neuroinflammation and contribute to behavioral and cognitive dysfunction [91].

Infiltrating cytokines such as interleukin-6 (IL-6) and interleukin-18 (IL-18) promote microglial activation toward a pro-inflammatory phenotype, thereby sustaining neuroinflammation and contributing to ongoing neuronal injury [72]. Additional pathways, including TWEAK/Fn14 signaling, have also been implicated in enhancing neuronal damage and vascular dysfunction, further aggravating central nervous system involvement [5,72].

In parallel, complement activation also plays a role in sustaining neuronal damage. C5a/C5aR signaling has been associated with increased oxidative stress, cytoskeletal alterations, and further disruption of endothelial integrity, reinforcing BBB dysfunction and promoting neuroinflammatory cascades [92].

At the clinical level, increased BBB permeability has been associated with higher disease activity indices and elevated markers of central nervous system injury, including glial fibrillary acidic protein (GFAP) and neurofilament light chain (NfL), supporting the link between barrier disruption and ongoing neurodegeneration [71,87].

### 5.5. Modulation by Cinnamon

*Cinnamomum cassia* has emerged as a potential modulator of inflammation, oxidative stress, and barrier dysfunction in lupus. Recent experimental works suggest that *Cinnamomum cassia* powder (as the powder of cinnamon bark with bark extract) may have neuroprotective effects in lupus models. In an imiquimod-induced lupus model, cinnamon demonstrated antioxidant and anti-apoptotic effects, including reduced oxidative stress and decreased neuronal apoptosis [15]. Complementary findings from a related animal study further showed that the administration of *Cinnamomum cassia* powder (as the powder of cinnamon bark and bark extract) was associated with the attenuation of behavioral abnormalities, alongside a reduction in neuroinflammatory markers and immune cell infiltration within the central nervous system [3]. These findings suggest that the protective effects of cinnamon may involve both systemic anti-inflammatory mechanisms and direct actions within the central nervous system (Table 2).

Experimental studies indicate that cinnamon-derived metabolites can exert central effects, including neuroprotective actions within the brain [106,107]. In addition, modeling studies have suggested that several cinnamon-derived compounds, including cinnamaldehyde, may cross the blood–brain barrier [103].

At the cellular level, cinnamon appears to modulate oxidative stress and apoptotic pathways within the brain. Alterations in redox homeostasis observed in lupus models have been shown to be attenuated following cinnamon administration, suggesting the preservation of oxidative balance. In addition, the modulation of apoptosis-related pathways, including FOXO3 and Bcl-2 signaling, supports a role for cinnamon in limiting neuronal injury and enhancing cellular resilience under inflammatory conditions [15].

Taken together, these findings suggest that the neuroprotective effects of *Cinnamomum cassia* in imiquimod-induced murine lupus may involve a dual mechanism, combining systemic immunomodulation with direct central nervous system actions. However, direct quantitative evidence linking cinnamon to changes in BBB permeability remains limited, and further studies are required to clarify its mechanisms of action and potential clinical relevance.

## 6. Future Perspectives: Integrating the Gut–Brain Axis

The dynamic between these two biological barriers in SLE and the modulating effect of cinnamon may be mediated by several hypothetical pathways that need to be confirmed in future studies.

The metabolite bridge may be linked to gut-derived short-chain fatty acids (SCFAs), which are frequently depleted in lupus patients [108]. These SCFAs are essential for maintaining the integrity of both barriers [109,110,111,112]. Their depletion favors a synchronized vulnerability: the gut becomes leakier while the BBB loses its ability to upregulate tight junction proteins like Claudin-5, as suggested in non-lupus studies [109]. Animal and human data suggest that cinnamon (especially polyphenol-rich extracts or essential oil) may support SCFA-producing bacteria and slightly increase certain SCFAs [113,114], possibly mediating the beneficial effect of cinnamon in lupus models.

Another probable bridge is zonulin, a central physiological modulator of intercellular TJs [115]. Elevated zonulin is emerging as a marker of leaky gut in SLE [116]. Higher zonulin levels are associated with measurable intestinal hyperpermeability, microbial dysbiosis, and the translocation of bacterial fragments into the systemic circulation, which may amplify systemic inflammation and autoimmunity in lupus [117]. In neuroinflammatory diseases, especially in multiple sclerosis-like models, zonulin elevation has been tied to increased BBB permeability and neurobehavioral changes [118]; this suggests a hypothetical pathway whereby gut-derived zonulin and inflammatory signals may interface with the CNS in SLE. Lupus-associated pathobionts like *Blautia gnavus* elevate zonulin, increasing gut permeability and allowing LPS and bacterial antigens to trigger hepatic and systemic TLR4/NF-κB signaling, potentiating the upregulation of inflammatory cytokines (IL-6, TNF-α) that cross or compromise the BBB [63]. While no study directly explores cinnamon’s effect on zonulin or its specific modification of *Blautia gnavus* expansion, the observed barrier-tightening, microbiota modulation and anti-inflammatory effects of cinnamon [3,75,113] may be consistent with a hypothetical pathway where cinnamon would counteract the zonulin-related loosening of tight junctions and possibly impact the gut–brain axis.

A third possible bridge between the gut barrier and BBB in lupus would be the kynurenine pathway. The kynurenine pathway is the main route of tryptophan metabolism, driven by enzymes such as indoleamine-2,3-dioxygenase-1 (IDO1), and producing immunomodulatory and neuroactive metabolites (e.g., kynurenine, kynurenic acid, quinolinic acid) [119]. Mouse and mechanistic work suggest that gut-derived inflammatory signals (LPS, pathobiont translocation) can induce IDO1 in dendritic cells and macrophages, thereby linking leaky gut directly to kynurenine-driven immunomodulation and potentially to neuropsychiatric manifestations [120,121,122]. While there are no direct experimental data showing that cinnamon specifically modulates the kynurenine pathway in humans or lupus, cinnamon’s broad anti-inflammatory, antioxidant, and immunomodulatory effects could be hypothetically linked to dampening kynurenin overactivation driven by inflammation and immune stress: cinnamon and its active components (polyphenols, cinnamaldehyde) suppress pro-inflammatory cytokines such as TNF-α, IL-6, and IFN-γ and inhibit NF-κB and MAPK signaling, all of which are upstream drivers of indoleamine-2,3-dioxygenase-1 (IDO1) [123].

Finally, Th17 cells are a key immune node along the gut–brain axis in non-lupus studies, linking gut microbiota signals to neuroinflammation, BBB disruption, and behavioral/cognitive changes, including depression-like and cognitive-impairment phenotypes, such as in experimental encephalomyelitis models [124,125]. A recent meta-analysis showed increased levels of Th17 cells and related cytokines correlated with elevated Th17/Treg ratios in SLE [126]. Cinnamon treatment suppressed Th1 and Th17 responses and augmented Th2 response in in vivo murine experimental encephalomyelitis [75]. Furthermore, cinnamaldehyde ameliorates ulcerative colitis through suppressing Th17 cells [127]. This cellular/cytokine bridge may be hypothesized to participate in the beneficial effect of cinnamon supplementation on the neuropsychiatric activity of lupus [3,15], and it deserves to be explored in future studies.

## 7. Conclusions

The intestinal barrier and BBB are emerging as key players in lupus pathogenesis, mediated by TJ alterations. As a multi-target natural compound, cinnamon has shown protective effects on these two barriers in imiquimod-induced murine lupus, with beneficial action on the gut–liver axis and neuropsychiatric manifestations. Future perspectives should reproduce and expand these effects in translational and pharmacological studies and build an integrated dynamic targeting *Cinnamomum cassia’s* modulation of the gut–brain axis in lupus.

## Figures and Tables

**Table 1:** Gut barrier, TJ dysfunction and associated pathological features in imiquimod-induced lupus and modulation by cinnamon [69,70]. Cinnamon powder (powder of cinnamon bark with bark extract) was offered individually to each animal on a small, flattened piece of chow, slightly moistened with water, on a daily basis.

Pathological Features	Lupus-Related Abnormalities	*Cinnamomum cassia* (200 mg/kg, 5 Days/Week) Effect
Intestinal Permeability	Significant decrease in critical tight junction proteins (Claudin, ZO-1, and Occludin). Early and persistent rise in fecal calprotectin (starting at day 22 through day 45).	Alleviated gut barrier degradation by protecting/modulating the expression of intestinal tight junction proteins (particularly Claudin and ZO-1), reducing overall permeability.
Gut Microbiota Alterations	Decline in the Firmicutes/Bacteroidota ratio. Decrease in Lactobacillaceae.	Mitigated the lupus-driven decline in the Firmicutes/Bacteroidota ratio. Exhibited a prebiotic-like effect by significantly increasing beneficial *Lactobacillus* and *Limosilactobacillus* abundances.
Bacterial Translocation	Increase in *E. coli* proteins in the liver.	Reversed the increase of *E. coli* protein in the liver.
Hepatic Inflammation Pathways	Marked activation of the TLR4, TLR7, and p-NFκB/NFκB inflammatory signaling pathways in liver tissue.	Normalized and downregulated the lupus-induced hepatic overexpression of TLR7 and p-NFκB/NFκB.
Hepatic Oxidative Stress	Liver tissue oxidative stress.	Successfully normalized the enhanced expression of SOD1 and SOD2 (superoxide dismutases).

**Table 2:** BBB, TJ dysfunction and associated pathological features in imiquimod-induced lupus and modulation by cinnamon. *Cinnamomum cassia* was used as powder (bark powder with bark extract).

Pathological Features	Lupus-Induced Abnormalities	Cinnamon Effect (*Cinnamomum cassia* Intervention) and Key Bioactive Compounds
Behavioral Cognitive Impairment	Depressive-like and anxiety behavior (assessed using the forced swim test and T-maze test). Impairment in spatial working memory and exploratory behavior (assessed using Y-maze alternation tests).	Reversed depressive-like and anxiety phenotypes and restored spatial working and exploratory memory capacity (especially effective when given as a preventive regimen) [3]. Key bioactive compounds implicated: Cinnamaldehyde, cinnamate, procyanidin [102,103].
Hippocampal histopathology	Caused severe neuronal shrinkage and nuclear chromatin condensation within the hippocampal cornu ammonis (CA) and dentate gyrus (DG) zones.	Exerted potent neuroprotection, lowering overall neurodegeneration scores, and preventing neuronal shrinkage and chromatin condensation within the CA and DG regions [3]. Key bioactive compounds implicated: cinnamaldehyde, sodium benzoate [104,105]
BBB	Destabilized BBB endothelial cells by altering the expression and tyrosine phosphorylation profiles of tight junction proteins (claudin-1, occludin, and ZO-1). Lupus mouse plasma induced cell–cell border delocalization of claudin-1, causing it to collapse into a punctate intracytoplasmic pattern.	Normalized the protein expression and physiological phosphorylation states of cerebral tight junctions of BBB [3]. Reversed claudin-1 delocalization, returning the protein to stable, linear cell-to-cell boundaries. Key bioactive compounds implicated: cinnamaldehyde, sodium benzoate, procyanidine [15,103,105].
Neuroinflammation	Triggered neuroinflammation via the activation of the TLR7–MyD88 pathway. Upregulated the NLRP3 inflammasome complex in the hippocampus.	Directly downregulated hippocampal TLR7 expression (supported by molecular modeling showing cinnamaldehyde binding/interacting with TLR7 at multiple docking sites) [3]. Attenuated neuroinflammation by lowering NLRP3 expression [3]. Key bioactive compounds implicated: cinnamaldehyde 2-methoxycinnamaldehyde, sodium benzoate [15,104,106].

## Data Availability

No new data were created or analyzed in this study. Data sharing is not applicable to this article.
